# Regression to Ambiguous Pain Classification by Introducing the Term ‘Mixed Pain’; A Commentary on Freynhagen et al. Regarding the Mixed Pain Concept

**DOI:** 10.1002/ejp.70386

**Published:** 2026-09-11

**Authors:** Eva Kosek

**Affiliations:** ^1^ Department of Clinical Neuroscience Karolinska Institutet Stockholm Sweden; ^2^ Department of Surgical Sciences, Clinical Pain Research Uppsala University, Uppsala University Hospital Uppsala Sweden

This journal recently published a paper by Freynhagen et al. (2026), entitled ‘Mixed Pain: Towards a Consensus Definition and a Mechanism‐Based Framework’, describing a structured consensus process based on Delphi methodology. A group of 14 experts, all required to have prior involvement in ‘mixed pain research’, participated. The procedure resulted in the definition of ‘mixed pain’ as: ‘pain that is associated with a lesion, disease, or disorder resulting in an overlap of at least two mechanistic pain descriptors (nociceptive, neuropathic, or nociplastic)’. The authors claim that this will: ‘help provide the context needed to align research, refine diagnosis and design mechanism‐based therapies for patients with mixed pain’ and that ‘it marks an important step towards conceptual clarification—from confusion to consensus, from overlap to action—transforming mixed pain from an ambiguous concept into a potential framework of modern pain taxonomy’.

However, despite these enthusiastic claims, there are several contradictions in the article, as well as problems with the term mixed pain. First, the authors state that mixed pain is a ‘distinct, clinical phenotype’, yet, the definition of mixed pain allows for four different phenotypes (nociceptive/neuropathic, nociceptive/nociplastic, neuropathic/nociplastic or all three).

Second, in the discussion, the authors recognize that ‘mixed pain does not represent a separate diagnostic category or a fourth mechanistic pain descriptor’ and that ‘identification of the contributing mechanistic pain descriptors remains essential and should accompany the use of the term whenever possible’, continuing that ‘mixed pain is not intended to serve as a label for unknown pain mechanisms’. However, as at least two mechanistic pain descriptors must be determined for a patient to fulfil the criteria of mixed pain, it is difficult to understand the meaning of ‘whenever possible’. Per definition it is not mixed pain unless the contributing mechanistic pain descriptors can be determined.

Furthermore, as identification of at least two specific mechanistic pain descriptors is mandatory, what additional information does the mixed pain term provide? What is the added value of saying that a patient suffering from moderate nociceptive back pain and severe neuropathic leg pain due to a lumbar disc herniation has mixed pain, in addition to describing the two pain types and their relative importance? This is never addressed in the paper. Instead, the authors argue that the term mixed pain may have therapeutic implications and potential clinical relevance, but without providing any evidence for this statement.

Finally, the authors propose that ‘mixed pain may represent more than a simple coexistence of mechanisms and reflect a distinct pattern of mechanistic interaction’. The notion that different pain types present simultaneously or concurrently in overlapping body regions interact is interesting and merits investigation. Yet, it is still hard to understand how the term would help to ‘align research, refine diagnosis and design mechanism‐based therapies for patients with mixed pain’, without distinguishing between the four different phenotypes within the mixed pain category.

The reason to classify pain into mechanistic categories is to reach a higher granularity allowing for choosing the best, most specific treatment options in the clinic or enhancing precision within research. If pain would be classified as purely nociceptive/neuropathic/nociplastic or mixed, precision would be lost. With all due respect, as clinicians are often pressed for time, it may become tempting to classify patients with mixed pain without taking the trouble to sort out the underlying distinct pain types and their relative importance, with detrimental effects for patient care.

In conclusion, either the term mixed pain must be accompanied by specific mechanistic pain descriptors, in which case it becomes redundant, or if used as a ‘separate category’ it denominates various heterogeneous combinations of mechanistic pain types obscuring mechanistic pain classification. The authors fail to discuss this dilemma and to provide convincing examples of how the use of the term mixed pain would provide added value in research or clinical practice.

## References

[ejp70386-bib-0001] Freynhagen, R. , B. Morlion , A. Alcántara Montero , et al. 2026. “Mixed Pain: Toward a Consensus Definition and a Mechanism‐Based Framework.” European Journal of Pain 30, no. 7: e70356. 42559696 10.1002/ejp.70356 PMC13445151

